# The Effects of an Indigenous Health Curriculum for Medical Students

**DOI:** 10.1007/s40670-020-00971-8

**Published:** 2020-05-08

**Authors:** Melissa E. Lewis

**Affiliations:** Department of Family and Community Medicine, University of Missouri School of Medicine, Columbia, MO USA

**Keywords:** Medical education, Medical curriculum, Indigenous health, Culturally appropriate care, Cultural intelligence, Ethnocultural empathy, Cultural humility, Social justice

## Abstract

**Purpose:**

Indigenous patients experience a variety of healthcare challenges including accessing and receiving needed healthcare services, as well as experiencing disproportionate amounts of bias and discrimination within the healthcare system. In an effort to improve patient-provider interactions and reduce bias towards Indigenous patients, a curriculum was developed to improve first-year medical students’ Indigenous health knowledge.

**Method:**

Two cohorts of students were assessed for their Indigenous health knowledge, cultural intelligence, ethnocultural empathy, and social justice beliefs before the lecture series, directly after, and 6 months later.

**Results:**

Results of paired *t* test analysis revealed that Indigenous health knowledge significantly improved after the training and 6 months later. Some improvements were noted in the areas of cultural intelligence and ethnocultural empathy in the second cohort.

**Conclusions:**

It is feasible to teach and improve Indigenous-specific health knowledge of medical students using a brief intervention of lectures. However, other critical components of culturally appropriate care including social justice beliefs and actions, ethnocultural empathy, and cultural humility may require increased and immersed cultural training.

## Introduction

“The ultimate aim of a curriculum on disparities is that learners develop a professional commitment to eliminating inequities in health care quality and understand and accept their role in eliminating racial and ethnic healthcare disparities [1].”

In the USA, Indigenous^1^ people receive poorer healthcare compared to non-Indigenous people and have worse health outcomes [2]. These disparities occur due to reduced access to primary care, use of lower-quality healthcare facilities, language barriers, and economic and educational disparities [3, 4]. In addition, ethnicity and race also play a role in the quality of care and treatment patients receive within the medical system [5]. When controlling for access-related factors, stereotyping, discrimination, and implicit bias are associated with health disparities for racial and ethnic minorities [6]. For instance, a survey of implicit bias among 154 providers found that one third agreed with negative stereotypes about Indigenous people; 84% both preferred non-Hispanic White patients to Indigenous patients and believed Indigenous children were more challenging and their caregivers less compliant compared to non-Hispanic White patients [7]. These biased beliefs have consequences for patients’ outcomes [8] and negatively affects patient safety [9]. From surgery outcomes [10] to maternal mortality rates [11] and for medical students [12], residents [13], surgeons [14], and nurses [15], race plays a role in adverse health outcomes.

About half of Indigenous people say that they experience racism very often [16]. In the medical setting in particular, Indigenous people are 2.6 times more likely to report racially based maltreatment [17] and are 10 times more likely to experience discrimination compared to non-Hispanic White patients [18]. A recent study that gathered experiences of Indigenous patients reported a variety of adverse experiences [8]. These patients often felt that they were experiencing bias, treated poorly, threatened, and dismissed. They felt that their medical problems were trivialized; that they were treated unkindly, especially in discussions regarding pain; and that these experiences led to worsened care. Racism is related to health disparities in the area of mental health, cancer, obesity, diabetes, heart disease, and even adverse birth outcomes [19–21].

The Institute of Medicine (IOM), the National Institutes of Health (NIH), and the Association of American Medical Colleges (AAMC) have all discussed the importance of providers and healthcare organizations providing culturally competent and equitable care [22–24]. Lack of attention to cultural factors in the clinic is related to a number of negative outcomes, including non-adherence, unnecessary visits and tests, and longer hospital stays [6]. Further, almost half of all providers (44%) say that addressing culture in clinic settings is “somewhat to extremely stressful,” [25] indicating a poor level of confidence and competence in the area that requires further training. However, there is evidence that training healthcare professionals about cultural issues can be effective in increasing knowledge on the topic [1] and improving health outcomes for racial and ethnic minority patients [26–28]. In a notable example, after providers at Massachusetts General Hospital received online cross-cultural training, the proportion of African-American patients who felt they received a lower quality of care than most White, English-speaking patients decreased from 25% in 2004 to 7% in 2012 [29].

While effective methods to improve cultural competency of healthcare providers exist, many challenges remain in delivery and effectiveness. Less than half of all fourth-year medical students demonstrate competency during common cross-cultural situations; the majority feel unskilled in providing basic cross-cultural care [30]. One partially successful medical-student training program aiming to prepare them for cross-cultural healthcare found improvements in assessment skills, collaboration with an interpreter, and level of preparedness in working with ethnic and cultural minority patients. However, the training did not change how students work with patients who use complementary and alternative medicine, have non-Western medicine health beliefs, distrust the medical system, are transgender patients, are religious minorities (including an inability to identify how religious beliefs affect clinical care and treatment), or are new immigrants [26]. Clearly some content and skills around cultural learning are more effectively acquired than others, demonstrating considerable room for improvement about how to teach this material effectively. Training carried out in classrooms, discussion groups, or with online learning methods might not be sufficient. Also, it is difficult to create and maintain a program around cultural effectiveness in a medical school despite the critical need for such programs [31]. In a review of five programs that implemented cultural competency curricula during residency, only 3 remain today [32]. However, even very little effort can increase learning in the area of cultural effectiveness and social justice. For example, two students at the John A. Burns School of Medicine at University of Hawaii at Manoa in the USA created and evaluated a program that taught medical students about social justice using online discussion groups and social media. They discovered that 92% of students increased knowledge of social justice [33]. This may be the first program that has successfully improved students’ social justice beliefs and has disseminated this information.

The Society for General Internal Medicine Health Disparities Task Force recommends three critical components for training in racial and cultural health disparities. It is important to address: (1) *attitudes* such as unconscious bias, stereotyping, and mistrust; (2) *knowledge* and history of health disparities using a social determinants of health lens; and (3) *skills* to effectively communicate, assess, and treat across cultures [1]. These components are important because, unfortunately, unconscious bias concerning patients’ race and social class plays a role in health professionals’ (including residents, surgeons, and nurses) decision-making [13]. Even providers who are aware of the social determinants of health rate patients’ health outcomes as being 90% attributable to patient factors, and over 40% did not believe there was strong evidence of the role of race in health outcomes [34]. Unconscious bias coupled with inaccurate beliefs of the cause of health outcomes for patients is magnified for Indigenous patients who experience prevalent stereotyping and bias within the healthcare system. Therefore, training that starts with basic information about the history of local communities, their political status, and cultural beliefs can help to reduce the perpetuation of false stereotypes that may contribute to unequal care.

The purpose of this project was to evaluate whether medical students who had received an Indigenous health lecture series at a single USA medical school had improved learning at the completion of the program and 6 months after the training. The hypothesis was that training would have a positive effect on students’ learning in the areas of knowledge, social justice, cultural empathy, cultural intelligence, and cultural humility at the completion of the program and at the follow-up period.

## The Intervention

First-year medical students in 2014 (year 1) and in 2015 (year 2) received 7 h of coordinated lectures over 1 to 2 weeks about Indigenous peoples’ history, culture, and health [35]. The majority of students (year 1 = 96.66%; year 2 = 88.33%) attended these lectures, as they are part of their core curriculum, yet attendance is not taken, and therefore, the courses are not mandatory. This content was delivered mainly by Indigenous faculty from the School of Medicine, American Indian Studies and Education. There were slight differences in the curriculum from year 1 to year 2 given faculty availability and updates that were made based on feedback from the faculty and students (see Appendix Table 4).

## Method

To evaluate the program, a one-group (non-randomized), pre-post-test quasi-experimental design was implemented. Students completed online questionnaires three times: 1 week before the lectures (pre-test), 2 weeks after the end of the lectures (post-test), and 6 months after the lectures (follow-up). However, in the year 1 post-test evaluation, four measures (standardized Social Justice Scale, Scale of Ethnocultural Empathy, Cultural Intelligence Scale, and Multigroup Ethnic Identity Measure) of the five total measures proved unavailable due to technical errors in the survey software, which prevented retrieving the data for analysis. The pre-test also requested demographic information, including age, gender, race/ethnicity, and current residence via zip code.

### Instruments

#### Indigenous Knowledge and Beliefs

To measure the content learning of participants, the author created the Indigenous knowledge and beliefs (IKB) scale. This scale was adapted from a lesson plan assessment developed by anti-racism educators at the University of Calgary [36], and the content was tailored to focus on the Indigenous people local to the region (see Appendix Table 5). The scale consists of ten short-answer items such as “Currently, how many federally recognized American Indian nations are there in Minnesota?” or “What was the main goal of the boarding school era?” Respondents could attempt to answer the question or select “I don’t know.” This measure has two summary scores: the Indigenous knowledge and beliefs summary score and the Indigenous knowledge and beliefs attempts summary score. For the Indigenous knowledge and beliefs summary score, possible individual item scores ranged from 0 to 1, with 0 indicating an incorrect answer and 1 indicating a correct answer. The 10 items were averaged to create an IKB summary score with higher scores indicating a higher knowledge of both local and broad Indigenous knowledge and more culturally appropriate beliefs.

In order to account for self-efficacy on the lecture topics, a calculated attempt (i.e., those individuals who responded anything other than “I don’t know”) score called the Indigenous knowledge and beliefs attempts summary score was also created using the average number of “attempts” whether they answered the question correctly or not. Individual item scores ranged from 0 to 1, with 0 indicating no attempt and 1 indicating an attempt.

#### Social Justice

The standardized Social Justice Scale (SJS) was used to capture medical student’s opinions about social justice (e.g., I feel confident in my ability to talk to others about social injustices and the impact of social conditions on health and well-being; I believe that it is important to respect and appreciate people’s diverse social identities) [36, 37]. This scale was designed to measure social justice–related values, attitudes, perceived behavioral control, subjective norms, and intentions based on a 4-factor conception of Ajzen’s theory of planned behavior. Confirmatory factor analysis and analyses for reliability and validity were used to test the properties of the original scale. Internal consistency was good for all four subscales: attitudes (*α* = .95), subjective norms (*α* = .82), perceived behavioral control (*α* = .84), and intentions (*α* = .88). The original measure was shortened from 29 items down to 17, which included the original 4 subscales (see Appendix Table 6).

#### Ethnocultural Empathy

Students were asked to answer 16 questions (e.g., I share the anger of those who face injustice because of their racial and ethnic backgrounds) from the Scale of Ethnocultural Empathy (ECE) that includes 4 scales: empathic feeling and expression, empathic perspective taking, acceptance of cultural differences, and empathic awareness [38]. This scale was shortened from 31 to 16 questions for relevancy and brevity (see Appendix Table 7). High internal consistency and test–retest reliability estimates of the original scale have been demonstrated [38].

#### Cultural Intelligence–Specified Indigenous Culture

Students were asked 10 items (out of 20 questions from the original measure; see Appendix Table 8) from a standardized scale examining cultural intelligence–specified for Indigenous culture (Cultural Intelligence Scale (CIS)) [39]. Items were on a 7-point Likert scale ranging from “1 = disagree strongly” to “7 = agree strongly” (e.g., I check the accuracy of my cultural knowledge as I interact with people from Indigenous cultures; I know the legal systems of Indigenous cultures). The original measure has demonstrated good reliability (*α* = .70–.86) and significant convergent and discriminant validity [39].

#### Multicultural Ethnic Identity

Students were asked to answer the 16-question Multigroup Ethnic Identity Measure (MEIM) [40]. Twelve items use a 4-point Likert scale (“1 = strongly agree” to “4 = strongly disagree”; e.g., I have a strong sense of belonging to my own ethnic group), and 4 items are open-ended and ask about cultural identity of self and of parents (e.g., my father’s ethnicity is?). Studies have demonstrated good reliability for both high school (*α* = .81) and college samples (*α* = .90) [40].

#### Analysis

Descriptive statistics were examined for all student characteristics including age, race/ethnicity, and gender. Additionally, paired *t* tests were conducted to examine changes in student responses between pre, post, and follow-up surveys for all study measures (i.e., Indigenous knowledge and beliefs, cultural intelligence–specified Indigenous knowledge, multicultural ethnicity identity, social justice, and ethnocultural empathy) with the exception noted above of missing data for four measures for the 2014 cohort post-test. Surveys for the three time points were linked using demographic information (birth date, zip code, gender), and a participant identification number was created. All analyses were performed in SAS, version 9.4 (SAS Institute, Cary, NC).

## Results

### Year 1

In year 1, there were a total of 60 students and 58 students (97%) filled out pre-lecture surveys; however, the number of participants that was used in the analysis is reduced given that the participants needed to have completed a survey in at least one other time period as is customary with paired *t* tests (see Table 2). Approximately half (55%) were male, 91% were White, and the mean age was 25.2 years (SD = 2.8); see Table 1. As shown in Table 2, students’ IKB summary scores increased significantly between pre- and post-lecture surveys (mean difference = 0.35, *p* < .0001) and between pre and follow-up (mean difference = 0.22, *p* < .0001). Student’s IKB summary scores, however, decreased significantly between post and follow-up (mean difference = − 0.09, *p* = .02). Student’s IKB summary attempt scores increased significantly between pre- and post-lecture surveys (mean difference = 0.42, *p* < .0001) and between pre and follow-up (mean difference = 0.36, *p* < .0001), but no differences were noted between post and follow-up (*p* = .69). Although student’s empathy, intelligence, and identity scores increased between pre and follow-up lecture, none were found to be statistically significant. Similarly, social justice scores decreased slightly between pre and follow-up, but the difference was non-significant.

**Table 1:** Sample demographics by year

	Year 1		Year 2	
	*n*	%	*n*	%
Total	58	–	53	–
Gender				
Male	32	55.2	24	45.3
Female	26	44.8	29	54.7
Race				
White/Caucasian	53	91.4	43	81.1
Native American/American Indian	3	5.2	4	7.5
Other race/unknown	2	3.4	6	11.3
Age, mean (SD)	25.2 (2.8)		23.8 (1.84)

**Table 2:** Student scores from year 1 surveys (pre, post, and follow-up)

	*n*	Pre	Post	Follow-up	*p* value
		Mean (SD)	Mean (SD)	Mean (SD)	
Indigenous knowledge and beliefs (IKB) summary					
Pre-post	41	0.11 (0.16)	0.46 (0.18)		< .0001
Post-follow-up	24		0.45 (0.20)	0.36 (0.18)	.02
Pre-follow-up	28	0.10 (0.16)		0.32 (0.19)	< .0001
IKB attempts					
Pre-post	46	0.17 (0.21)	0.59 (0.25)		< .0001
Post-follow-up	33		0.56 (0.25)	0.54 (0.27)	.69
Pre-follow-up	34	0.16 (0.22)		0.52 (0.29)	< .0001
Empathy (ECE)					
Pre-follow-up	32	5.15 (0.72)		5.26 (0.70)	.21
Intelligence (CIS)					
Pre-follow-up	32	4.85 (0.96)		5.08 (0.94)	.06
Social justice (SJS)					
Pre-follow-up	32	6.08 (0.48)		6.03 (0.52)	.69
Identity (MEIM)					
Pre-follow-up	34	2.75 (0.41)		2.80 (0.47)	.33

Data collection periods: post, 2 weeks; follow-up, 6 months. Numbers in each comparison are less than the overall sample size as only those who completed surveys at both periods could be included in comparisons. Significance is denoted by *p* < .05

*SD* standard deviation, *ECE* Scale of Ethnocultural Empathy, *CIS* Cultural Intelligence Scale, *SJS* Social Justice Scale, *MEIM* Multigroup Ethnic Identity Measure

### Year 2

In year 2, there were a total of 60 students, of which 53 students (88%) filled out pre-lecture surveys and fewer represented in the analysis (see Table 3). Slightly less than half (45%) of the students were male, 81% were White, and the mean age was 23.8 years (SD = 1.8); see Table 1. Similar to year 1, student’s IKB summary scores at year 2 (Table 3) increased significantly between pre- and post-lecture surveys (mean difference = 0.15, *p* < .0001) and between pre and follow-up (mean difference = 0.09, *p* < .001). The decrease in IKB summary scores between post and follow-up was not significant (*p* = .11). Students’ IKB summary attempt scores at year 2 also increased significantly between pre- and post-lecture surveys (mean difference = 0.34, *p* < .0001) and between pre and follow-up (mean difference = 0.21, *p* < .0001). A significant decrease between student’s post and follow-up IKB summary attempt scores was noted (*p* = .02).

**Table 3:** Student scores from year 2 surveys (pre, post, and follow-up)

	*n*	Pre	Post	Follow-up	*p* value
		Mean (SD)	Mean (SD)	Mean (SD)	
Indigenous knowledge and beliefs (IKB) summary					
Pre-post	40	0.17 (0.22)	0.32 (0.30)		< .0001
Post-follow-up	30		0.32 (0.30)	0.26 (0.26)	.11
Pre-follow-up	40	0.17 (0.16)		0.26 (0.26)	< .001
IKB attempts					
Pre-post	40	0.29 (0.26)	0.63 (0.25)		< .0001
Post-follow-up	30		0.64 (0.25)	0.53 (0.27)	.02
Pre-follow-up	40	0.33 (0.29)		0.54 (0.28)	< .0001
Empathy (ECE)					
Pre-post	36	4.93 (0.64)	5.17 (0.71)		.0004
Post-follow-up	22		5.14 (0.64)	4.97 (0.75)	.03
Pre-follow-up	31	4.98 (0.67)		4.99 (0.71)	.88
Intelligence (CIS)					
Pre-post	40	4.47 (0.83)	5.49 (0.53)		< .0001
Post-follow-up	30		5.50 (0.55)	5.00 (0.71)	< .0001
Pre-follow-up	40	4.61 (0.92)		5.07 (0.67)	.0006
Social justice (SJS)					
Pre-post	40	6.09 (0.72)	6.05 (0.84)		.66
Post-follow-up	30		5.98 (0.93)	6.08 (0.89)	.13
Pre-follow-up	40	6.12 (0.69)		6.14 (0.82)	.79
Identity (MEIM)					
Pre-post	40	2.15 (0.43)	2.07 (0.43)		.05
Post-follow-up	30		2.09 (0.47)	2.11 (0.67)	.91
Pre-follow-up	40	2.06 (0.47)		1.97 (0.68)	.38

Data collection periods: post, 2 weeks; follow-up, 6 months. Numbers in each comparison are less than the overall sample size as only those who completed surveys at both periods could be included in comparisons. Significance is denoted by *p* < .05

*SD* standard deviation, *ECE* Scale of Ethnocultural Empathy, *CIS* Cultural Intelligence Scale, *SJS* Social Justice Scale, *MEIM* Multigroup Ethnic Identity Measure

Student’s empathy (ECE) scores at year 2 (Table 3) increased significantly between pre- and post-lecture surveys (mean difference = 0.24, *p* < .0001) but not between pre and follow-up (mean difference = 0.01, *p* = .88), whereas post-ECE scores were significantly higher than follow-up ECE scores (mean difference = 0.17, *p* = .03). Student intelligence (CIS) post and follow-up scores were significantly higher than pre-lecture scores (pre-post mean difference = 1.02, pre-follow-up mean difference = 0.46, *p* < .001), while post-CIS scores were significantly higher than follow-up CIS scores (mean difference = 0.50, *p* < .0001). No significant differences were noted in student’ social justice and identity scores between pre, post, or follow-up lecture surveys.

## Discussion

This project tested the effects of adding seven didactic hours to a first-year medical school curriculum mostly delivered by Indigenous faculty. The curriculum was nested in a university with a mission of American Indian Health and a Center for American Indian Minority Health, which houses the American Indian medical student recruitment core.

Our results suggest that Indigenous health lectures have lasting effects on students’ knowledge up to at least 6 months. Knowledge is the first component in most models of cultural effectiveness, dexterity, and humility. In fact, three recommendations to address structural racism in medicine include the following: “First, learn about, understand, and accept the United States’ racist roots. Second, understand how racism has shaped our narrative about disparities. Third, define and name racism [41].” Similarly, in an international consensus statement around medical education, colonization, racism, and privilege were named as the strongest barriers to Indigenous health [42]. Authors expressed the importance of decolonizing western medical education through changes in the medical infrastructure led by Indigenous people.

The study intervention, however, had less strong of an effect on students’ growth in other domains. For instance, cultural intelligence was approaching significance in year 1 from pre-test to follow-up (mean difference = 0.23, *p* = .06) and was significant in year 2 from pre-test to follow-up (mean difference = 0.46, *p* = .006), indicating that there was a trend of improvement in this measure. While student’s ethnocultural empathy (ECE) scores at year 1 did not reach a level of significance, in year 2 (Table 2), ECE increased significantly between pre- and post-lecture surveys (mean difference = 0.24, *p* < .0001) but not between pre and follow-up (mean difference = 0.01, *p* = .88); see Table 3. Although there were improved effects, possibly due to updated content in the second year, the intervention did not have long-lasting effects on student’s empathy. This is interesting because students continued to maintain their knowledge about Indigenous people, which, one would think, would affect beliefs and, therefore, empathy, yet it did not in the long term. It is possible that although one learns and continues to retain the information, the effect of actively learning and discussing the topic affects empathy, but not in isolation and not over time. It is also possible that the dose was insufficient for lasting effects, which requires more testing.

Other programs that have been successful at improving the knowledge, skills, and beliefs of students around Indigenous health have added additional components besides didactic learning including experiential learning opportunities. For instance, the University of Arkansas Medical Sciences Northwest campus created an interdisciplinary program to address health disparities with a focus on Marshallese patient populations [43]. This program was comprised of two didactic lectures co-created by a Native Marshallese physician, an experiential component within the clinic, and a service learning project within the community. Students who participated in this program reported significantly improved cultural competence and readiness for interpersonal learning, in addition to improved knowledge, skills, and behaviors towards working with Marshallese patients. At the John A. Burns School of Medicine, Native Hawaiian specific cultural competence training began in 2003 and now electives include a Dean’s certificate in Hawaiian Native Health as well as the Kalaupapa service learning project [44–46]. Evaluations of this program indicated that over 95% of medical students in the Native Hawaiian health elective either agreed or strongly agreed that they have a better understanding of tools that can be used for Native Hawaiian people to heal from cultural trauma. Students also reported that they learned about Indigenous perspectives and beliefs and it reinforced their commitment to rural and underserved areas [45]. It appears that experiential learning opportunities, whether in clinic or in community, provide a boost to the effects of teaching didactic content resulting in improved readiness and skills to work with Indigenous patients.

In the case of social justice beliefs and cultural humility in this study, neither was significantly improved at post-test or follow-up. These domains appear more difficult to change than knowledge and skills. However few, there are programs that have had success in teaching social justice, which may provide strategic examples for other developing medical curriculums. In particular, at the University of Otago in New Zealand, a study was conducted to assess social accountability within Indigenous medical education. Social accountability is a similar concept to social justice. Stakeholders including students, teachers, and community members were interviewed about how this Indigenous curriculum created social accountability to address Indigenous health disparities. They reported that they believed that the following components of the curriculum created social accountability for health professionals: (1) advocacy for Māori health in the community, university, and clinics; (2) putting social engagement into practice; and (3) creating a transformative practice around advocating for Māori health [47]. The University of Otago puts social accountability into practice through its commitment to address Māori health disparities mandated through the Treaty of Waitangi, using a strategic plan to recruit Māori students and teaching students more Indigenous health content than any other university in the world [48]. Through qualitative interviews, researchers discovered that this Indigenous health curriculum taught students how to advocate for patients and become change agents to reduce Indigenous health disparities. In other words, this curriculum has resulted in improved social justice beliefs and actions of students. In this case, a government decree, recruitment efforts, and a larger quantity of hours taught about Indigenous health resulted in improvements in social justice beliefs.

Although not Indigenous focused, a preclinical social justice program at Mount Sinai requires “a didactic course, faculty and student mentorship, research projects in social justice, longitudinal policy and advocacy service projects, and a career seminar series [49].” Students were interviewed in regard to their learning from this program, and the most common theme was that students were able to apply their learning about health disparities and social determinants of health to clinical encounters. This is an important outcome and highlights the need to teach social justice in a variety of ways (e.g., didactic, research project, and service project) with mentorship for both students and faculty.

In searching for best practices to improve social justice, an area that did not see improvement in our study, it is important to look to other programs that have had long-standing missions around social justice to guide future projects given the lack of evaluation research in this area. In particular, at the University of Hawaii at Manoa, core competencies in medical social justice cover knowledge, skills, and beliefs regarding social determinants of health, health justice advocacy, and community needs [50]. The purpose of this social justice program is to implement a student-driven curriculum to expose learners to various components of social justice in health and medicine by using their own inputs for content and design [51]. At the Geisel School of Medicine at Dartmouth, a social justice expert group convened that led to curriculum revision and improved student competencies in social justice [52]. The addition of student-led curriculum and social justice competencies creates a bar for all students to meet and also requires more faculty to put social justice content into their lectures [53] which may prove to increase the social justice competency of students.

### Limitations

The purpose of the research project was to test the learning of medical students before and after they received a series of seven lectures. No other program in the USA has implemented Indigenous health content within the core curriculum for all students and tested it. Therefore, the first goal of this investigation was to determine if the training was feasible and to test the hypothesis that training has a positive effect on students’ learning in the areas of knowledge, social justice, cultural empathy, cultural intelligence, and cultural humility. A one-group (non-randomized), pre-post-test was implemented using a quasi-experimental design and a convenience sample of first-year medical students. The author recognizes that with this design, there are substantial threats to both internal and external validity given the selection of participants and the lack of control groups and randomization. Measures were condensed in this study to reduce respondent fatigue. Due to modifications to some of the measures, it is possible that the reliability and consistency of these measures were not maintained. Further, while our team tried to make improvements to the lecture during year 2, small differences to the intervention exist and therefore cohorts 1 and 2 did not receive the exactly the same intervention. A power analysis was not completed before the data was analyzed. Given this limitation, some of the analyses may have lacked power possibly explaining some of the null results.

This intervention included a lecture series created by local stakeholders and was tailored to represent tribes of the region and may not be representative to other communities and tribes. For instance, stakeholders were very outspoken about the needs of the local Indigenous community and what they wanted their healthcare professionals to know about them, which may not be the case in all other communities. It is important to note with over 570 tribes in the USA alone, one must not generalize and assume similarities exist between them.

### Implications

There are many barriers for effective healthcare for Indigenous people. Without a medical provider who knows about your culture and health beliefs available at your local clinic, or an administrator that advocates for your community, healthcare is unlikely to effectively service Indigenous patients in that community. According to the AAMC, in 2015, only 0.2% of US medical school (not including osteopathic medicine programs) applicants were Indigenous, only 21 of 19,553 US medical student graduates in 2017 were Indigenous, only 0.6% are active physicians, and only 0.5% are full-time faculty [54]. Further, Indigenous representation in medical education has decreased, not increased, in the USA over the last several years and there are very few Indigenous faculty in medical schools (0.1%) or in the sciences [55]. Fewer underrepresented potential faculty are being hired as assistant professors when compared to those from well-represented groups, and simulation models suggest that this inequity will not change for the next 60 years at least [56]. Therefore, more needs to be done to include Indigenous students and faculty in the medical field. Content important to underrepresented groups should be reflected in the curriculum; however, this cannot be done without Indigenous advocates and allies.

### Future Directions

The solution to addressing Indigenous health disparities is multifaceted and requires intervention at multiple points. Intentionality is important. Programs that set out to teach values and beliefs around patient-centeredness and social justice advocacy have succeeded in reaching these goals [57]. For example, to improve social justice beliefs and behaviors, successful programs create core competencies and train faculty. Further, medical school initiatives and missions focused around underserved populations increase the number of providers practicing in those areas [58]. It is important to be intentional and strategic about the goal of addressing health disparities and also address the root of the problem. For deep structural changes to happen with regard to Indigeneity, decolonizing approaches could address rebalancing of systems of hierarchy in educational systems, critically analyzing the role of colonization in medicine and healthcare, and addressing root causes of disparate outcomes like racism and inequity. Although the Indigenous curriculum in this study resulted in some positive learning outcomes, future projects will work to be more holistic in nature including engaging more Indigenous medical education experts, more patient voices, and more administrative collaboration, as well as work to create learning objectives, core competencies, and a variety of and an increased amount of learning opportunities such as mentoring, service learning, and student-led initiatives.

## Conclusion

Culturally appropriate care in the USA is critical to the improved health of Indigenous people and requires a trained health workforce. This study found that medical student knowledge could be persistently increased by a lecture series, but other key values were less strongly affected. Future work should aim to discover what types of interventions (e.g., didactic vs. experiential), what amount, and at what intervals of training will produce significant improvements in healthcare providers’ knowledge, beliefs, and skills. At this intersection of health justice and education justice, Indigenous health content is critical; it is especially important for programs located on or near communities with higher rates of Indigenous patients who can work to reduce health disparities directly.

## Appendix

**Table 4:** Timing of lectures 2014 and 2015

2014	2015	Lecture	Lecturer(s)
x	x	Brief History of Native People	Erik Redix
x	x	Sovereignty and Politics	Joseph Bauerkemper
x	x	Culture, Spirituality and Resiliency	Brian McInnes
x	x	IHS	Dr. Deanna Dick (2014); Dr. Lorraine Turner (2015)
x	x	Medical Racism, Unconscious Bias, Cultural Humility & Self-Assessment	Melissa Lewis and Jill Doerfler (2014); Melissa Walls (2015)
	x	Indigenous Identity & Belonging	Jill Doerfler (2015)
x		Panel with Student Questions	Melissa Lewis, Jill Doerfler, Roxanne Gould, and Mary Owen (2014)
	x	Where do I go now? Techniques of Cultural Humility and Reducing Bias for Physicians	Jim Allen, Emily Onello, and Melissa Walls (2015)

Timing of 2014 lectures occurred from October 14 to October 24. Timing of 2015 lectures occurred from October 13 to 16

**Table 5:** Indigenous knowledge and beliefs item differences

Question	2014	2015
1	Please list Indigenous groups that have lived or currently live in the city you reside in	Please list Indigenous groups that have lived or currently live in the city you reside in
2	Currently, how many tribes are there in Minnesota?	Currently, how many tribes are there in Minnesota?
3	Currently, how many federally recognized American Indian nations are there in the USA?	Currently, how many federally recognized American Indian nations are there in the USA?
4*	What did the American Indian Removal Act seek to accomplish?	What was the main goal of the termination era?
5*	What are 3 types of land on Indian Reservations?	How many reservations are there in Minnesota?
6	What was the main goal of the federal boarding schools for American Indians?	What was the main goal of the federal boarding schools for American Indians?
7	Please (a) list 3 words from an Indigenous language and (b) state the language you will be using	Please (a) list 3 words from an Indigenous language and (b) state the language you will be using
8	The USA and American Indian nations have a __________ relationship	The USA and American Indian nations have a __________ relationship
9	Indian Health Service has how many regions in the USA?	Indian Health Service has how many regions in the USA?
10	Give 2 primary aspects of American Indian identity	Give 2 primary aspects of American Indian identity

All were open-ended questions with an “I do not know” option as well

*The two questions were changed between years 1 and 2 to match changes to the lecture

**Table 6:** Social Justice Scale (original and modified)

	Subscale	Item
1	Social Justice Attitudes	I believe that it is important to make sure that all individuals and groups have a chance to speak and be heard, especially those from traditionally ignored or marginalized groups*
2	Social Justice Attitudes	I believe that it is important to allow individuals and groups to define and describe their problems, experiences, and goals in their own terms*
3	Social Justice Attitudes	I believe that it is important to talk to others about societal systems of power, privilege, and oppression
4	Social Justice Attitudes	I believe that it is important to try to change larger social conditions that cause individual suffering and impede well-being*
5	Social Justice Attitudes	I believe that it is important to help individuals and groups to pursue their chosen goals in life
6	Social Justice Attitudes	I believe that it is important to promote the physical and emotional well-being of individuals and groups
7	Social Justice Attitudes	I believe that it is important to respect and appreciate people’s diverse social identities*
8	Social Justice Attitudes	I believe that it is important to allow others to have meaningful input into decisions affecting their lives*
9	Social Justice Attitudes	I believe that it is important to support community organizations and institutions that help individuals and group achieve their aims*
10	Social Justice Attitudes	I believe that it is important to promote fair and equitable allocation of bargaining powers, obligations, and resources in our society*
11	Social Justice Attitudes	I believe that it is important to act for social justice
12	Social Justice Perceived Behavioral Control	I am confident that I can have a positive impact on others’ lives
13	Social Justice Perceived Behavioral Control	I am certain that I possess an ability to work with individuals and groups in ways that are empowering*
14	Social Justice Perceived Behavioral Control	If I choose to do so, I am capable of influencing others to promote fairness and equality
15	Social Justice Perceived Behavioral Control	I feel confident in my ability to talk to others about social injustices and the impact of social conditions on health and well-being*
16	Social Justice Perceived Behavioral Control	I am certain that if I try, I can have a positive impact on my community*
17	Social Justice Subjective Norms	Other people around me are engaged in activities that address social injustices*
18	Social Justice Subjective Norms	Other people around me feel that it is important to engage in dialogue around social injustices
19	Social Justice Subjective Norms	Other people around me are supportive of efforts that promote social justice*
20	Social Justice Subjective Norms	Other people around me are aware of issues of social injustices and power inequalities in our society*
21	Social Justice Behavioral Intentions	In the future, I will do my best to ensure that all individuals and groups have a chance to speak and be heard*
22	Social Justice Behavioral Intentions	In the future, I intend to talk with others about social power inequalities, social injustices, and the impact of social forces on health and well-being*
23	Social Justice Behavioral Intentions	In the future, I intend to engage in activities that will promote social justice*
24	Social Justice Behavioral Intentions	In the future, I intend to work collaboratively with others so that they can define their own problems and build their own capacity to solve problems*

*Seventeen items were selected for this study

**Table 7:** Scale of Ethnocultural Empathy (original and modified)

	Subscale	Item
1	Empathic Feeling and Expression	When I hear people make racist jokes, I tell them I am offended even though they are not referring to my racial or ethnic group*
2	Empathic Feeling and Expression	I do not care if people make racist statements against other racial or ethnic groups
3	Empathic Feeling and Expression	I rarely think about the impact of a racist or ethnic joke on the feelings of people who are targeted*
4	Empathic Feeling and Expression	When other people struggle with racial or ethnic oppression, I share their frustration
5	Empathic Feeling and Expression	I feel supportive of people of other racial and ethnic groups, if I think they are being taken advantage of
6	Empathic Feeling and Expression	I share the anger of those who face injustice because of their racial and ethnic backgrounds*
7	Empathic Feeling and Expression	I share the anger of people who are victims of hate crimes (e.g., intentional violence because of race or ethnicity)
8	Empathic Feeling and Expression	When I know my friends are treated unfairly because of their racial or ethnic backgrounds, I speak up for them
9	Empathic Feeling and Expression	I get disturbed when other people experience misfortunes due to their racial or ethnic backgrounds
10	Empathic Feeling and Expression	I am touched by movies or books about discrimination issues faced by racial or ethnic groups other than my own*
11	Empathic Feeling and Expression	When I see people who come from a different racial or ethnic background succeed in the public arena, I share their pride*
12	Empathic Feeling and Expression	I am not likely to participate in events that promote equal rights for people of all racial and ethnic backgrounds*
13	Empathic Feeling and Expression	I seek opportunities to speak with individuals of other racial or ethnic backgrounds about their experiences
14	Empathic Feeling and Expression	When I interact with people from other racial or ethnic backgrounds, I show my appreciation of their cultural norms
15	Empathic Feeling and Expression	I express my concern about discrimination to people from other racial or ethnic groups*
16	Empathic Perspective Taking	It is easy for me to understand what it would feel like to be a person of another racial or ethnic background other than my own
17	Empathic Perspective Taking	It is difficult for me to relate to stories in which people talk about racial or ethnic discrimination they experience in their day-to-day lives
18	Empathic Perspective Taking	It is difficult for me to put myself in the shoes of someone who is racially and/or ethnically different from me
19	Empathic Perspective Taking	I know what it feels like to be the only person of a certain race or ethnicity in a group of people
20	Empathic Perspective Taking	I can relate to the frustration that some people feel about having fewer opportunities due to their racial or ethnic backgrounds*
21	Empathic Perspective Taking	I feel uncomfortable when I am around a significant number of people who are racially/ethnically different than me*
22	Empathic Perspective Taking	I do not know a lot of information about important social and political events of racial and ethnic groups other than my own*
23	Acceptance of Cultural Differences	I feel irritated when people of different racial or ethnic backgrounds speak their language around me
24	Acceptance of Cultural Differences	I feel annoyed when people do not speak standard English*
25	Acceptance of Cultural Differences	I get impatient when communicating with people from other racial or ethnic backgrounds, regardless of how well they speak English*
26	Acceptance of Cultural Differences	I do not understand why people want to keep their Indigenous racial or ethnic cultural traditions instead of trying to fit into the mainstream*
27	Acceptance of Cultural Differences	I do not understand why people of different racial or ethnic backgrounds enjoy wearing traditional clothing
28	Empathic Awareness	I am aware of how society differentially treats racial or ethnic groups other than my own*
29	Empathic Awareness	I recognize that the media often portrays people based on racial or ethnic stereotypes*
30	Empathic Awareness	I can see how other racial or ethnic groups are systematically oppressed in our society
31	Empathic Awareness	I am aware of institutional barriers (e.g., restricted opportunities for job promotion) that discriminate against racial or ethnic groups other than my own*

*Sixteen items were selected for this study

**Table 8:** Cultural Intelligence Scale (original and modified)

	Subscale	Item
1	Motivational Cultural Intelligence: Drive	I enjoy interacting with people from different cultures*
2	Motivational Cultural Intelligence: Drive	I am confident that I can socialize with locals in a culture that is unfamiliar to me*
3	Motivational Cultural Intelligence: Drive	I am sure I can deal with the stresses of adjusting to a culture that is new to me
4	Motivational Cultural Intelligence: Drive	I enjoy living in cultures that are unfamiliar to me
5	Motivational Cultural Intelligence: Drive	I am confident that I can get accustomed to the shopping conditions in a different culture
6	Cognitive Cultural Intelligence: Knowledge	I know the legal and economic systems of other cultures*
7	Cognitive Cultural Intelligence: Knowledge	I know the rules (e.g., vocabulary, grammar) of other languages*
8	Cognitive Cultural Intelligence: Knowledge	I know the cultural values and religious beliefs of other cultures*
9	Cognitive Cultural Intelligence: Knowledge	I know the marriage systems of other cultures
10	Cognitive Cultural Intelligence: Knowledge	I know the arts and crafts of other cultures
11	Cognitive Cultural Intelligence: Knowledge	I know the rules for expressing non-verbal behaviors in other cultures
12	Metacognitive Cultural Intelligence: Strategy	I am conscious of the cultural knowledge I use when interacting with people with different cultural backgrounds
13	Metacognitive Cultural Intelligence: Strategy	I adjust my cultural knowledge as I interact with people from a culture that is unfamiliar to me*
14	Metacognitive Cultural Intelligence: Strategy	I am conscious of the cultural knowledge I apply to cross-cultural interactions
15	Metacognitive Cultural Intelligence: Strategy	I check the accuracy of my cultural knowledge as I interact with people from different cultures*
16	Behavioral Cultural Intelligence: Action	I change my verbal behavior (e.g., accent, tone) when a cross-cultural interaction requires it*
17	Behavioral Cultural Intelligence: Action	I use pause and silence differently to suit different cross-cultural situations*
18	Behavioral Cultural Intelligence: Action	I vary the rate of my speaking when a cross-cultural situation requires it
19	Behavioral Cultural Intelligence: Action	I change my non-verbal behavior when a cross-cultural situation requires it*
20	Behavioral Cultural Intelligence: Action	I alter my facial expressions when a cross-cultural interaction requires it

Anywhere the word “culture” was used in an item, the word was changed to “Indigenous cultures”

*Ten items were selected for this study

## Abbreviations

AAMC: Association of American Medical Colleges
CIS: Cultural Intelligence Scale
ECE: Scale of Ethnocultural Empathy
IKB: Indigenous knowledge and beliefs
IOM: Institute of Medicine
MEIM: Multigroup Ethnic Identity Measure
NIH: National Institutes of Health
SJS: Social Justice Scale

## Footnotes

1 In this manuscript, the term *Indigenous* refers to Native people worldwide. Other commonly employed terms for Indigenous people in North America have included American Indian, Native American, Alaska Native, Native Hawaiian, and First Nations. The word Indigenous will be capitalized in this manuscript to indicate that this group is distinct from other racial and ethnic groups and sovereign nations. Although not always possible within this manuscript, it is important to use local and tribal language when referring to specific tribes whenever possible.

## References

[1] Smith WR, Betancourt JR, Wynia MK, Bussey-Jones J, Stone VE, Phillips CO, Fernandez A, Jacobs E, Bowles J (2007). Recommendations for teaching about racial and ethnic disparities in health and health care. Ann Intern Med.

[2] Lewis ME, Myhra LL (2018). Integrated care with Indigenous populations: considering the role of health care systems in health disparities. J Health Care Poor Underserved.

[3] Hasnain-Wynia R, Kang R, Landrum MB, Vogeli C, Baker DW, Weissman JS (2010). Racial and ethnic disparities within and between hospitals for inpatient quality of care: an examination of patient-level hospital quality Alliance measures. J Health Care Poor Underserved.

[4] Commonwealth Fund. A review of the quality of health care for American Indians and Alaska Natives. Commonwealth Fund. 2004. https://www.commonwealthfund.org/publications/fund-reports/2004/sep/review-quality-health-care-american-indians-and-alaska-natives. Accessed Oct 5 2017.

[5] Johnson RL, Saha S, Arbelaez JJ, Beach MC, Cooper LA. Racial and ethnic differences in patient perceptions of bias and cultural competence in health care. 2004. doi:10.1111/j.1525-1497.2004.30262.x, 19, 101, 110. 10.1111/j.1525-1497.2004.30262.x PMC1492144 15009789

[6] Nelson AR, Stith AY, Smedley BD (2003). Unequal treatment: confronting racial and ethnic disparities in health care.

[7] Puumala SE, Burgess KM, Kharbanda AB, Zook HG, Castille DM, Pickner WJ, Payne NR (2016). The role of bias by emergency department providers in care for American Indian children. Med Care.

[8] Goodman A, Fleming K, Markwick N, Morrison T, Lagimodiere L, Kerr T (2017). “They treated me like crap and I know it was because I was Native”: the healthcare experiences of Aboriginal peoples living in Vancouver's inner city. Soc Sci Med.

[9] Walker R, St Pierre-Hansen N, Cromarty H, Kelly L, Minty B (2010). Measuring cross-cultural patient safety: identifying barriers and developing performance indicators. Healthcare Quarterly (Toronto, Ont).

[10] Goyal MK, Kuppermann N, Cleary SD, Teach SJ, Chamberlain JM (2015). Racial disparities in pain management of children with appendicitis in emergency departments. JAMA Pediatr.

[11] Louis JM, Menard MK, Gee RE (2015). Racial and ethnic disparities in maternal morbidity and mortality. Obstet Gynecol.

[12] Haider AH, Efron DT, Swoboda S, Villegas CV, Haut ER, Bonds M (2011). Association of unconscious race and social class bias with vignette-based clinical assessments by medical students. JAMA..

[13] Haider AH, Schneider EB, Sriram N, Dossick DS, Scott VK, Swoboda SM, Losonczy L, Haut ER, Efron DT, Pronovost PJ, Lipsett PA, Cornwell EE, MacKenzie EJ, Cooper LA, Freischlag JA (2015). Unconscious race and social class bias among acute care surgical clinicians and clinical treatment decisions. JAMA Surgery.

[14] Haider AH, Schneider EB, Sriram N, Dossick DS, Scott VK, Swoboda SM, Losonczy L, Haut ER, Efron DT, Pronovost PJ, Freischlag JA, Lipsett PA, Cornwell EE, MacKenzie EJ, Cooper LA (2014). Unconscious race and class bias: its association with decision making by trauma and acute care surgeons. J Trauma Acute Care Surg.

[15] Haider AH, Schneider EB, Sriram N, Scott VK, Swoboda SM, Zogg CK (2015). Unconscious race and class biases among registered nurses: vignette-based study using implicit association testing. J Am Coll Surg.

[16] Yamashiro A, Goodyear-Ka'opua N (2014). Achieving social and health equity in Hawaii.

[17] Larson A, Gillies M, Howard PJ, Coffin J (2007). It's enough to make you sick: the impact of racism on the health of Aboriginal Australians. Aust N Z J Public Health.

[18] Harris R, Tobias M, Jeffreys M, Waldegrave K, Karlsen S, Nazroo J (2006). Effects of self-reported racial discrimination and deprivation on Māori health and inequalities in New Zealand: cross-sectional study. Lancet.

[19] Kaholokula JK, Iwane MK, Nacapoy AH (2010). Effects of perceived racism and acculturation on hypertension in Native Hawaiians. Hawaii Med J.

[20] Kaholokula JKA, Alvarez AN, CTH L, Neville HA (2016). Racism and physical health disparities. The cost of racism for people of color: contextualizing experiences of discrimination. Cultural, racial, and ethnic psychology book series.

[21] Kaholokula JKA, Antonio MCK, Ing CKT, Hermosura A, Hall KE, Knight R, et al. The effects of perceived racism on psychological distress mediated by venting and disengagement coping in Native Hawaiians. BMC Psychology. 2017. 10.1186/s40359-017-0171-6. 10.1186/s40359-017-0171-6 PMC5228113 28081710

[22] Crossing the quality chasm: a new health system for the 21st century. Washington, D.C.: National Academy Press; 2001. 25057539

[23] Association of American Medical Colleges. Cultural competence education. 2005. https://www.aamc.org/download/54338/data/culturalcomped.pdf. Accessed Oct 15 2017. 10.1097/00001888-200507000-00019 15980092

[24] Kagawa-Singer M, Dressler WW, George SM, Elwood WN (2015). The cultural framework for health: an integrative approach for research and program design and evaluation.

[25] De Souza IET, editor. Intercultural mediation in healthcare: myths and facts. Cross Cultural Health Conference; 2017 February 17, 2017; Honolulu, Hawaii.

[26] Weissman JS, Betancourt J, Campbell EG, Park ER, Kim M, Clarridge B, Blumenthal D, Lee KC, Maina AW (2005). Resident physicians' preparedness to provide cross-cultural care. JAMA: Journal of the American Medical Association.

[27] Massachusets General Hospital, Center for Diversity and Inclusion. Cross-cultural education. 2017. http://www.massgeneral.org/mao/multiculturaled/. Accessed Oct 15 2017.

[28] Watt K, Abbott P, Reath J (2016). Developing cultural competence in general practitioners: an integrative review of the literature. BMC Fam Pract.

[29] Betancourt JR, editor. Cross-cultural training for health professionals. Cross Cultural Health Conference; 2017 February 17, 2017; Honolulu, Hawaii.

[30] Green AR, Chun MBJ, Cervantes MC, Nudel JD, Duong JV, Krupat E, Betancourt JR (2017). Measuring medical students’ preparedness and skills to provide cross-cultural care. Health Equity.

[31] Chun MBJ, Takanishi DM (2009). The need for a standardized evaluation method to assess efficacy of cultural competence initiatives in medical education and residency programs. Hawaii Med J.

[32] Shah SS, Sapigao FB, Chun MBJ (2017). An overview of cultural competency curricula in ACGME-accredited general surgery residency programs. J Surg Educ.

[33] Porter T, Jones E, editors. Student engagement with health disparities through Facebook and video chat. Cross Cultural Health Conference; 2017 February 17, 2017; Honolulu, Hawaii.

[34] Britton BV, Nagarajan N, Zogg CK, Selvarajah S, Schupper AJ, Kironji AG (2016). Awareness of racial/ethnic disparities in surgical outcomes and care: factors affecting acknowledgment and action. Am J Surg.

[35] Lewis M, Prunuske A (2017). The development of an Indigenous health curriculum for medical students. Acad Med.

[36] Chagnon-Greyeyes C, Johnston B, Kelly J, Paquette D, Srivastava A, Wong T. Indigenous knowledge handout. CARED Collective, Calgary. 2015. http://www.ucalgary.ca/cared/indigenousknowledgehandout. Accessed Mar 13 2019.

[37] Torres-Harding SR, Siers B, Olson BD (2012). Development and psychometric evaluation of the Social Justice Scale (SJS). Am J Community Psychol.

[38] Wang Y-W, Davidson MM, Yakushko OF, Savoy HB, Tan JA, Bleier JK (2003). The Scale of Ethnocultural Empathy: development, validation, and reliability. J Couns Psychol.

[39] Ang S, Dyne L, Koh C. Development and validation of the CQS. Routledge; 2008.

[40] Phinney JS (1992). The Multigroup Ethnic Identity Measure: a new scale for use with diverse groups. J Adolesc Res.

[41] Hardeman RR, Medina EM, Kozhimannil KB (2016). Structural racism and supporting black lives - the role of health professionals. N Engl J Med.

[42] Jones, R., Crowshoe, L., Reid, P., Calam, B., Curtis, E., Green, M., ... & Milroy, J. (2019). Educating for indigenous health equity: an international consensus statement. Acad Med, 94(4): 512–519. 10.1097/ACM.0000000000002476 PMC6445615 30277958

[43] McElfish PA, Moore R, Long CR, Purvis RS, Rowland B, Buron B (2017). Integrating interprofessional education and cultural competency training to address health disparities. Teach Learn Med.

[44] Gaddis C, editor. The dean’s certificate in Native Hawaiian Health. Cross Cultural Health Conference; 2017; Honolulu, Hawaii.

[45] Lee WK, Harris CCD, Mortensen KA, Long LM, Sugimoto-Matsuda J (2016). Enhancing student perspectives of humanism in medicine: reflections from the Kalaupapa service learning project. BMC Med Educ.

[46] Carpenter D-AL, Kamaka ML, Kaulukukui CM (2011). An innovative approach to developing a cultural competency curriculum; efforts at the John A. Burns School of Medicine, Department of Native Hawaiian Health. Hawaii Med J.

[47] Pitama, S., Beckert, L., Huria, T., Palmer, S., Melbourne-Wilcox, M., Patu, M., … & Wilkinson, T. J. (2019). The role of social accountable medical education in addressing health inequity in Aotearoa New Zealand. J R Soc New Zealand, 1–14

[48] Pitama S. (2013) “As natural as learning pathology” the design, implementation and impact of indigenous health curricula within medical schools. Doctoral dissertation. University of Otago.

[49] Bakshi S, James A, Hennelly MO, Karani R, Jakubowski A, Ciccariello C (2015). The Human Rights and Social Justice Scholars Program: a collaborative model for preclinical training in social medicine. Ann Glob Health.

[50] Schiff T, Rieth K (2012). Projects in medical education: “Social Justice in Medicine” a rationale for an elective program as part of the medical education curriculum at John A. Burns School of Medicine. Hawaii J Med Public Health.

[51] Ambrose AJ, Andaya JM, Yamada S, Maskarinec GG (2014). Social justice in medical education: strengths and challenges of a student-driven social justice curriculum. Hawaii J Med Public Health..

[52] Coria A, McKelvey TG, Charlton P, Woodworth M, Lahey T (2013). The design of a medical school social justice curriculum. Acad Med.

[53] Hixon AL, Yamada S, Farmer PE, Maskarinec GG (2013). Social justice: the heart of medical education. Soc Med.

[54] American Association of Medical Colleges. Total U.S. medical school graduates by race/ethnicity and sex, 2013-2014 through 2017-2018. 2018. https://www.aamc.org/download/321536/data/factstableb4.pdf. Accessed Oct 15, 2019

[55] American Association of Medical Colleges. Distribution of U.S. medical school faculty by sex and race/ethnicity. 2016. https://www.aamc.org/download/475556/data/16table8.pdf. Accessed Oct 15 2017

[56] Gibbs KD, Basson J, Xierali IM, Broniatowski DA. Decoupling of the minority PhD talent pool and assistant professor hiring in medical school basic science departments in the US. eLife. 2016;5. 10.7554/eLife.21393. 10.7554/eLife.21393 PMC5153246 27852433

[57] Cha SS, Ross JS, Lurie P, Sacajiu G (2006). Description of a research-based health activism curriculum for medical students. J Gen Intern Med.

[58] Mullan F, Chen C, Petterson S, Kolsky G, Spagnola M (2010). The social mission of medical education: ranking the schools. Ann Intern Med.

